# Transient Increase in Cortical Excitability Following Static Stretching of Plantar Flexor Muscles

**DOI:** 10.3389/fphys.2018.00530

**Published:** 2018-06-11

**Authors:** Francesco Budini, Monica Christova, Eugen Gallasch, Paul Kressnik, Dietmar Rafolt, Markus Tilp

**Affiliations:** ^1^Institute for Sport Science, Graz University, Graz, Austria; ^2^Otto Loewi Research Center, Physiology Section, Medical University of Graz, Graz, Austria; ^3^Institute of Physiotherapy, Institute of Applied Sciences FH-Joanneum, Graz, Austria; ^4^Center for Medical Physics and Biomedical Engineering, Medical University of Vienna, Vienna, Austria

**Keywords:** spinal reflexes, cortical excitability, static stretching, motor evoked potentials, H-reflex

## Abstract

Spinal excitability in humans is inhibited by both passively holding a static position with the muscle lengthened (static stretching) and by a single non-active lengthening movement. However, whilst immediately after a passive lengthening movement the inhibition persists for several seconds, there seem to be an immediate recovery following static stretching. This result is counter intuitive and could be attributed to methodological procedures. Indeed, differently to what has been done until now, in order to study whether static stretching has a transient effect on the neuromuscular pathway, the procedure should be repeated many times and measurements collected at different time points after stretching. In the present study we repeated 60 times 30 s static stretching of ankle plantar flexors and measured tap reflex (T-reflex), Hoffman reflex (H-reflex), and motor evoked potentials (MEPs) from the Soleus muscle at several time points, starting from immediately after until 30 s following the procedure. T-reflex was strongly inhibited (range 31–91%, *p* = 0.005) and the inhibition persisted for 30 s showing a slow recovery (*r* = 0.541, *p* = 0.037). H-reflex was not affected by the procedure. Stretching increased the size of the MEPs (*p* < 0.0001), differences at times 0 and 2 s after stretching (*p* = 0.015 and *p* = 0.047, respectively). These results confirm that static stretching reduces muscle spindle sensitivity. Moreover it is suggested that post-activation depression of Ia afferents, which is commonly considered the cause of H-reflex depression during both dorsiflexion and static stretching, vanished immediately following stretching or is counteracted by an increased corticospinal excitability.

## Introduction

Conditioning a muscle by means of stretching induces changes not only in muscle compliancy (Konrad et al., 2017), but also in discharge and sensitivity of muscle spindles (Gregory et al., 1987), excitability of spinal pathways (Budini et al., 2017), and cortical excitability (Stuart et al., 2002). However, from a neuromuscular point of view there seems to be a noticeable difference between conditioning the muscle through static stretching (passively holding a static position with the muscle lengthened) and through a passive eccentric movement (single non-active lengthening movement). Indeed, at the static end position of a stretch, H-reflex (Robinson et al., 1982; Etnyre and Abraham, 1986; Guissard et al., 1988, 2001; Voigt and Sinkjær, 1998; Hwang, 2002; Masugi et al., 2017), stretch and tap reflex (Robinson et al., 1982; Guissard et al., 1988) and motor evoked potentials (Guissard et al., 2001) are inhibited. Surprisingly though, after static stretching is applied, regardless either its duration (Opplert et al., 2016) or intensity (Guissard et al., 2001; Masugi et al., 2017), as soon as the joint angle is returned to its anatomical zero position, no acute neurological alteration is commonly reported with the exception of tap and stretch reflex inhibition (Avela et al., 1999; Budini et al., 2017). However, tap and stretch reflex inhibition are attributed to a reduction of muscle spindle sensitivity imputed to formation of some slack in the intrafusal muscle fibers (Proske et al., 1993), therefore this neurological response seems to be the consequence of a mechano-morphological change. Differently to traditional static stretching, conditioning a muscle through a passive eccentric movement consists in either elongating the muscle a single time and returning it to its neutral anatomical position (e.g., dorsiflex the ankle joint and back) or in reaching the neutral anatomical position with an eccentric movement (starting for example with the ankle joint in plantar flexion position). Following this procedure, spinal excitability was reported to be strongly inhibited for several seconds (2–15 s) (Delwaide and Hugon, 1969; Nielsen et al., 1993, 1995; Hultborn et al., 1996; Wood et al., 1996; Voigt and Sinkjær, 1998).

By looking at the existing literature, it remains unclear how the considerable neural changes observed during static stretching and after a single passive eccentric movement are not present after static stretching. The most probable explanation is that normal conditions are promptly restored within few seconds; therefore, the time window to look at changes is narrow. In fact, as highlighted in a recent literature review (Budini and Tilp, 2016), a common limitation of those studies that investigated neural changes following a period of static stretching, is that the values were collected starting from immediately after the procedure, with an appropriate delay from one stimulation and the next, until a sufficient number of values were collected (Mark et al., 1968; Guissard et al., 1988, 2001; Vujnovich and Dawson, 1994; Yapicioglu et al., 2013). This means that what is reported to be the result immediately after stretching is in reality an average value that represents the situation within one or more minutes after stretching. On the contrary, for assessing the effect of a single passive eccentric movement, data were collected always at the same time point after the movement and, in order to have an appropriate number of values to average, the movement was repeated several times (Nielsen et al., 1993, 1995; Hultborn et al., 1996; Wood et al., 1996; Voigt and Sinkjær, 1998).

Therefore, for evaluating the effect of stretching on parameters such as cortical and spinal excitability that need several averaged recordings for obtaining a reliable value and that have a rapid recovery time, the stretching procedure should also be repeated many times. The aim of the present work is therefore to address this procedural pitfall and investigate the acute effect of stretching on spinal and cortical excitability. By applying this methodology we expect to observe an inhibition in both H-reflex and MEPs lasting several seconds similar to that reported in those studies where single dorsiflexion movements were repeated. Moreover, by comparing the changes in H-reflex, T-reflex, and MEPs we should be able to locate the origin of the inhibition at spinal or cortical level.

## Methods

### Ethical approval

The study was conformed to the standards set by the Declaration of Helsinki and approved by the local research ethics board (Ethikkommission der Karl-Franzens-Universität Graz). Written informed consent was obtained from all volunteers before the onset of the experimental procedures.

### Participants

Fourteen recreationally active sport science students (5 male age 23.6 ± 1.5 years, body mass 75.2 ± 7.3 kg, stature 180.8 ± 7.9 cm, and 9 female age 24.3 ± 2.0 years, body mass 58.0 ± 3.0 kg, stature 166.1 ± 4.8 cm) with no history of neurological disorders volunteered for the experiment. Volunteers were required to abstain from any strenuous physical activity on the testing day as well as to refrain from taking caffeine-containing substances and smoking within 2 h before the testing session.

### Study design

Participants attended the laboratory on two separate occasions: the first, lasting about 90 min, for familiarizing with testing procedures and equipment and the second for the actual testing session. Testing session lasted about 3 h.

The experiment consisted in the measurement of H-reflex, T-reflex, and MEPs after transcranial magnetic stimulation (TMS) before and at different time points following stretching.

Ten TMS stimulations were perform at each of the following time points after stretching: 0, 2, 4, 5, 7, 9, 10, 12, 14, 15, 17, 19, 20, 22, 24, 25, 27, 29, 30, 32 s. Six H-reflexes stimulations were performed at each of 0, 2, 4, 6, 8, 10, 12, 14, 16, 18, 20, 22, 24, 26, 28 s after stretching. Six T-reflexes were performed at each of 1, 3, 5, 7, 9, 11, 13, 15, 17, 19, 21, 23, 25, 27, 29 s after stretching. Different stimulation types (H-reflex, T-reflex, and TMS) did not overlap, meaning for instance that the sequence that induced the stimulation at time 0 s for H-reflex was a different sequence that the one inducing stimulation at time 0 for TMS. Stimulations were distributed over several attempts also to maintain an appropriate inter-stimulus interval. For example the stimulation sequence that activated the H-reflex at time 0 s, activated the same reflex again only at time 10 and 20 s after stretching. A different sequence was used for time 2, 12, 22 s after stretching and so on.

### Experimental procedures

Subjects were sitting on an isokinetic dynamometer (CON-TREX MJ, CMV AG, Duebendorf, Switzerland) with the standard setup for ankle joint movement individually adjusted. Participants had their right knee fully extended and the foot resting on the dynamometer footplate, the ankle joint aligned with the dynamometer rotation shaft and the ankle angle set at 10° plantar flexion deviating from a neutral position at 90°. Volunteers sat with the trunk at 110° and the head supported by a cushion (dentafix®, pro medico Handels GmbH, Graz, Austria) that once positioned could be deflated allowing the formation of a stable form molded on the volunteers' head and neck shapes. By using a remote control, the volunteers were instructed to adjust the dorsiflexion isokinetic rotation operated by the dynamometer around the foot plate until the point of perceived maximal dorsiflexion. Participants were asked to keep their knee extended and to relax during the procedures.

Once the maximal individual dorsiflexion was defined, subjects left the dynamometer and were prepared for electromyographic recording (EMG) recording from tibialis anterior (TA) and soleus (SOL) muscles. Subsequently, the volunteers sat down again on the dynamometer chair (position as described above), stimulation intensity for TMS was determined and two complete H-M stimulation ramps were collected.

Volunteers were then instructed to relax completely and either keep their eyes closed or gaze at a 4 m distance point. A trigger-driven sequence of stimulations involving T-reflex, H-reflex, and TMS started and continued for 150 s until 15 T-reflexes, 15 H-reflexes, and 30 MEPs were collected for baseline reference values.

After baseline recordings, the foot was passively rotated at 20°/s to the full dorsiflexion angle defined at beginning of the testing session, for two times 30 s with no rest in between except for the time needed to return to 10° plantar flexion (PF) position and back again to maximal dorsiflexion position. Immediately after the ankle returned to the 10° PF angle following the second bout of stretching (between 1.9 and 2.6 s depending on the angle reached for static stretching), a sequence of stimulations started and continued for 30 s. Following these 30 s, the subject was instructed to make a short (2 s) isometric contraction of the plantar flexors, at circa 10% of maximal strength (with the aim of regaining slack in muscle spindle fibers through alpha-gamma co-activation), and was then allowed to remove the foot from the foot plate for the time needed to organize another stretch bout (about 20 s). Subsequently, the volunteer repositioned the foot on the foot plate and the two times 30 s stretch was repeated with another sequence of stimulations triggered at the end of the stretching. Eight different sequences were used in random order to collect data at each time point after stretching. For example sequence “One” triggered the TMS at time after stretching 0, 5, 10, 15, 20, 25, 30′′; H-reflex at time 2, 12, and 22′′ after stretching and T-reflex at 7, 17, and 27′′ after stretching. Sequence “One” was used six times and then a new sequence was uploaded in order to obtain another four TMS stimulations at the above listed time points and to test other time points for H- and T-reflexes. The 2 X 30′′ stretches were repeated 30 times, in this way for each selected time point after stretching 10 MEPs, 6 H-reflexes, and 6 T-reflexes were collected.

### Surface electromyography

Volunteers were prepared for surface EMG from the SOL and TA of the right leg, and from the tibialis anterior of the left leg. Recordings from the left leg were only used to ensure the correct directional positioning of the TMS coil. Electrodes (Blue Sensor N, Ambu A/S, Ballerup, Denmark) for recording H-reflex from the SOL muscle were placed in monopolar configuration (as suggested by Hadoush et al., 2009); all the other electrodes were placed in standard bipolar configuration at an interelectrode distance of 20 mm. Two ground electrodes (on per leg) were placed over the tibial bone medial surface. The gain for the EMG signal was 180.

### Stimulations

All stimulations were performed with the ankle joint at 10° PF.

H and M waves measured in SOL were elicited by electrical stimulation (KeyPoint® 2-channel) delivered to the tibial nerve by rectangular pulses of 1.0 ms duration. The anode (5 × 9 cm, STIMEX adhesive gel electrode) was placed on the patellar tendon and the cathode was placed in the popliteal fossa overlying the nerve at a position that provided the greatest H wave amplitude at the smallest stimulus intensity possible. Before baseline recordings two complete ramps (from H onset to the 120% stimulation intensity needed to elicit an M_max_) were collected. The stimulation intensity was then adjusted to obtain a value at which the H wave was still in its ascending phase and an M wave was visible (this stimulation intensity was usually close to the H_max_, and corresponded on average to 4.9% of the M_max_). This intensity was then used throughout the experiment and the current delivered by the stimulator was adjusted when needed to ensure constant amplitude of the M wave (Aagaard et al., 2002).

Tendon tap reflex was elicited by a motor (Type GDRX 075, Magnet-Schultz, Germany) driven hammer hitting the Achilles tendon about 3–4 cm above its insertion on the calcaneus. An electrical output from the motor provided information about its rotation allowing hammer displacement and acceleration to be monitored.

Motor evoked potentials in response to single pulse TMS were recorded from SOL and TA of the right leg. TMS was performed with Magstim 200, (Magstim Company Ltd., UK) using a double cone coil (110 mm coil diameter). The coil was placed over the M1 of the leg area, 1–2 cm posterior from the vertex and slightly rotated to the left side in order obtain the largest response from the contralateral right SOL. Resting motor threshold was determined as the minimum stimulator intensity able to evoke MEPs in SOL of at least 50 μV amplitude in more than 50% out of 10 consecutive trials (Rossini et al., 1994). To ensure a constant coil positioning throughout the experiments, subjects were wearing EEG caps where the optimal coil position was marked with a soft pen. Then 30 MEPs were elicited at 5-s intervals with stimulation intensity equal to 120% of the resting motor threshold.

### Data analysis

Electromyography, foot displacement, trigger and motor output signals were synchronized (DEWESoft® 7.0 recording system, DEWETRON GmbH, Austria), digitized with a sampling frequency of 10 KHz, and stored on a PC. In order to avoid phase shift no low pass filter was applied. Limitation of the bandwidth with 60 kHz was determined by the isolation amplifier. No aliasing effect was observed. Data was analyzed using custom algorithms developed in Matlab (R2014b).

The 15 H waves recorded at baseline were checked for consistency and those related to an M wave showing peak to peak amplitude exceeding the target stimulation intensity by ±2 standard deviations were discarded (Budini et al., 2017). Intensity of stimulation of the 90 post stretch measurements (6 stimulations for each of the 15 time points investigated) was also checked for consistency in relation to the size of the M waves.

Baseline T-reflex waves and MEPs with peak to peak amplitude exceeding by ±2 standard deviations the average baseline value were discarded (Nogueira-Campos et al., 2014). Similarly, consistency was checked within the 90 T-reflexes (6 stimulations, 15 time points) and 200 MEPs (10 stimulations, 20 time points) collected post stretching. All the remaining waves were retained for statistical analysis.

### Statistical analysis

Measurements were checked for normal distribution by Shapiro–Wilk test.

Changes in spinal excitability were assessed by comparing the H/M ratio at baseline and at the post-stretching time points with a Friedman test.

MEPs and T-reflexes values at each time point and at baseline were normalized by the M_max_ and analyzed with an ANOVA for repeated measurements (or Friedman for data not normally distributed). In case of significant result, paired *T*-test (or Wilcoxon signed ranks test) was used for pair comparisons vs. baseline. Pearson's correlation was used for linear regression analysis (T-reflex and MEPs TA), Spearman's rho for non-parametric regression analysis of MEPs SOL. All statistical analysis was completed using PASW Statistic 18.0.0.

## Results

### T-reflex

**Figure 1:**
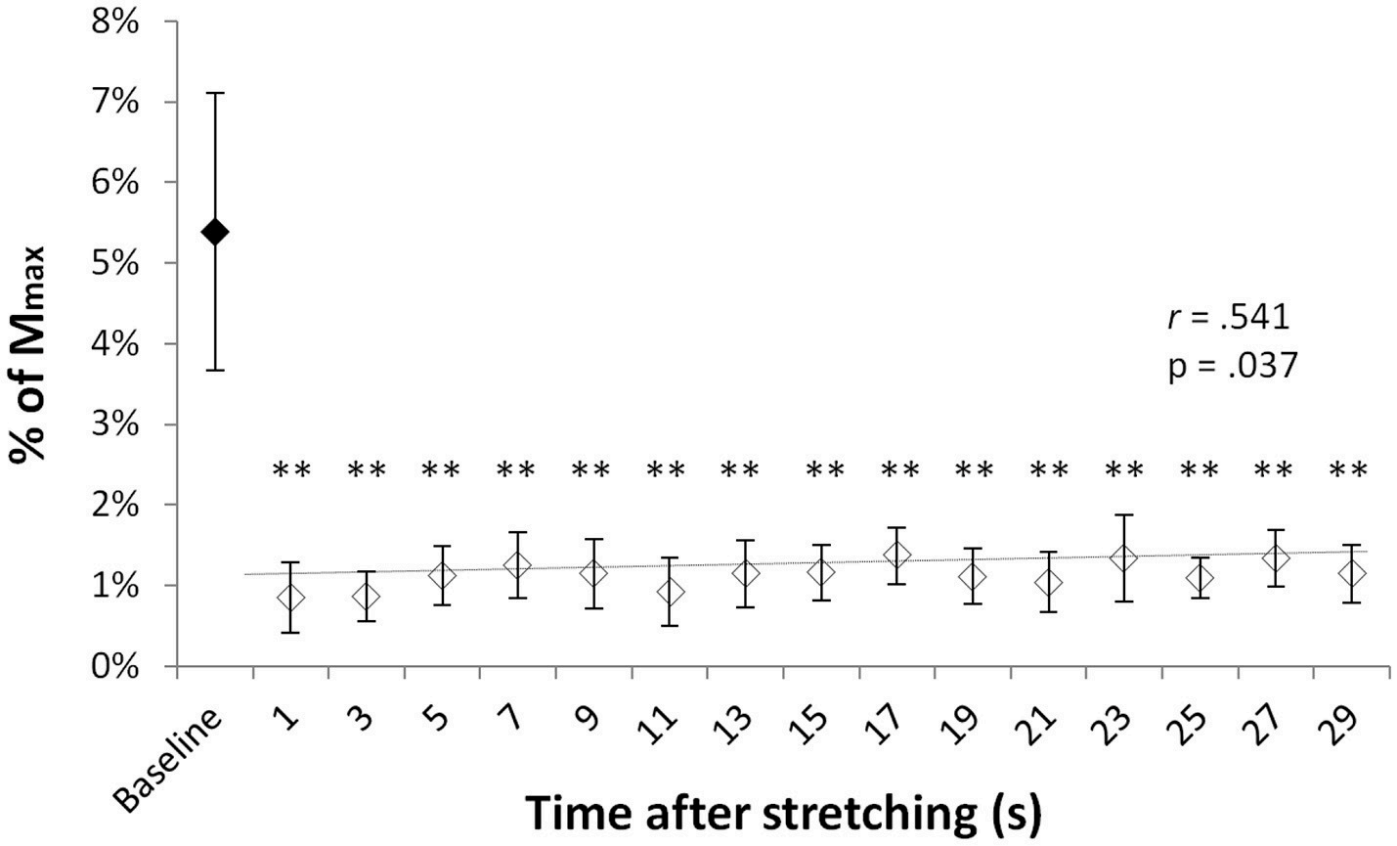
T-reflexes as percentage of the M_max._ Diamonds represent group mean ± *SD* of T-reflex peak to peak expressed as % of the M_max_ at baseline (black diamonds) and at every investigated time point after stretching (white diamonds). The trend line (baseline value excluded) shows the reflex recover during the 29 s following stretching. ^**^p < 0.01.

T-reflex could be found in all but one volunteer. Group average T-reflexes at baseline corresponded to 4.8 ± 3% of the M_max_, with an absolute peak to peak amplitude ranging from 0.246 to 2.76 mV. Following stretching there was a considerable reduction in reflex amplitude (range 31–93%) in respect to baseline values, with significantly different group average T-reflex values ranging from 0.85 and 1.37% of the M_max_ (minimum and maximum values observed at time points 0′′ and 17′′, respectively). Figure 1 shows the group average size of the T-reflex expressed as percentage of the M_max_ for baseline and each of the 15 time points after stretching. Stretching had significant effect on T-reflex [$\chi_{\left(3\right)}^{2}$ = 34.493, *p* = 0.005] with reflexes measured at baseline larger than any other reflex after stretching. Values showed a slow but significant recover throughout the 30 s after stretching as shown by a positive correlation between time after stretching and T-reflex amplitude (*r* = 0.541, *p* = 0.037, *b* = 0.0002).

### H-reflex

**Figure 2:**
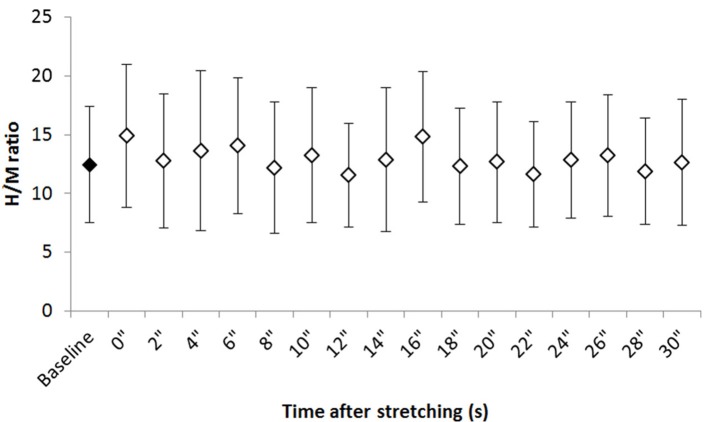
Group average H/M ratio at baseline and after stretching. Group average ±*SD* for H/M ratio at baseline (black diamond) and each time point following stretching (white diamonds).

Figure 2 shows the group average H/M ratio at baseline and at every investigated time point after stretching. Despite most of the time points after stretching show an higher H/M ratio compared to baseline, Friedman test revealed that stretching did not influence this parameter [$\chi_{\left(3\right)}^{2}$ = 24.906, *P* = 0.07].

### MEPs

**Figure 3:**
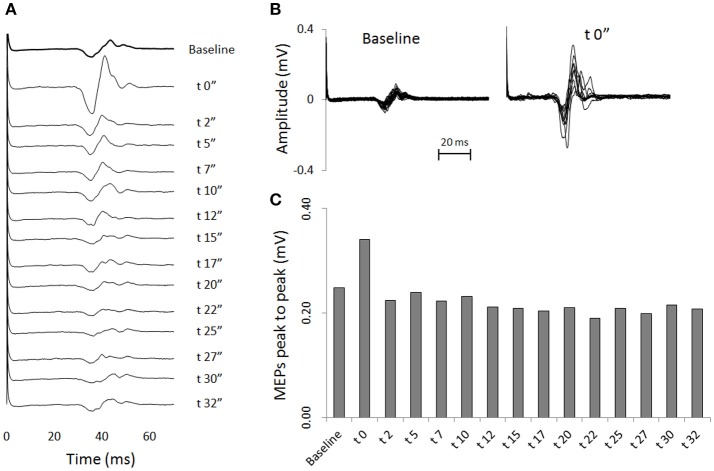
One subject sample MEPs. For a representative subject: **(A)** MEPs are shown as raw EMG signals for baseline and for 14 time points after stretching. Each line is the average of 20 (baseline) or 10 (after stretching) measurements. **(B)** Superimposed EMG tracks of MEPs of baseline measurements (20 tracks) and measurements at time post 0′′ (10 tracks). **(C)** Average amplitude peaks to peak of the MEPs waveforms.

**Figure 4:**
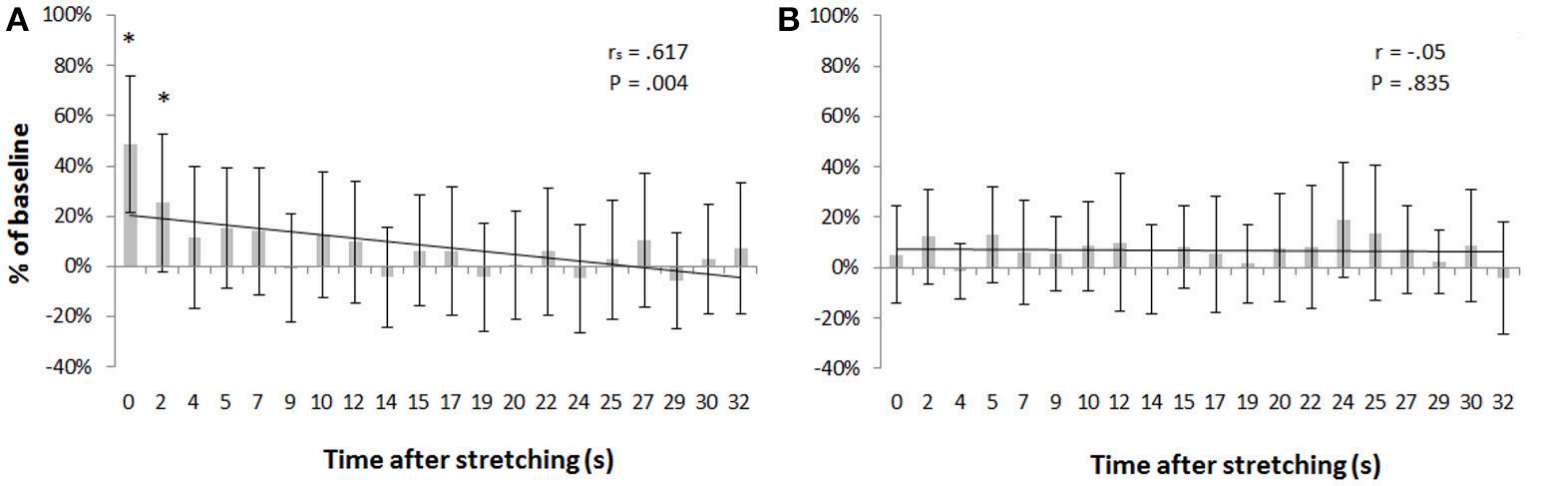
MEPs amplitude as percentage of baseline. Bars represent group average ±*SD* of MEPs expressed as percentage of baseline for each of the 20 time points after stretching. **(A)** SOL muscle, **(B)** TA muscle. ^*^p < 0. 05.

Transcranial magnetic stimulation was well tolerated by all participants and no side-effects were reported. The mean resting motor threshold was 46.25 ± 9.25% of the stimulator output. Figure 3 shows, for a representative participant, the amplitude of the MEPs at baseline and 14 other time points after stretching. A clear increase can be seen at time 0′′ (Figures 3A,B), for then returning close to baseline value (Figure 3C). Group average results confirm that stretching increased the size of the MEPs (*F* = 3.8383, *p* < 0.0001), differences were found between baseline and measurements after stretching at times 0′′ and 2′′ (*p* = 0.015 and *p* = 0.047, respectively). There was a negative correlation between the time after stretching and the MEPs amplitude (*r*_s_ = 0.617, *p* = 0.004; Figure 4A). No differences were observed between baseline and post stretching at any time point for the TA muscle (Figure 4B).

## Discussion

The present experiment confirms some of the known effects of muscle stretching on spinal reflex excitability and reveals a previously unreported effect on corticospinal excitability. The study demonstrates for the first time that MEPs are facilitated immediately after static stretching.

### H-reflex

It is commonly reported, that H-reflex is reduced during static stretching (Robinson et al., 1982; Etnyre and Abraham, 1986; Voigt and Sinkjær, 1998; Guissard et al., 2001; Hwang, 2002; Masugi et al., 2017), however, following this procedure, H reflex amplitude is re-established as soon as the foot returns to neutral joint position (Guissard et al., 1988, 2001; Vujnovich and Dawson, 1994; Yapicioglu et al., 2013; Opplert et al., 2016; Masugi et al., 2017). Measurements of spinal and cortical excitability are subjected to large spontaneous fluctuations between trials, for this reason it is necessary to collect and average several values and also allow a sufficient inter-stimuli interval between two consecutive stimulations (Zehr, 2002; Misiaszek, 2003). The need of repeating the stimulations several times with the obligation of respecting an appropriate inter-stimuli interval, likely compromise the possibility of detecting a variation if this has a natural quick recovery and the measurements are collected following a single conditioning procedure. To address this methodological pitfall, in the present experiment stretching was repeated several times and values collected at the same time points after the intervention.

It has been suggested that during static stretching both pre- and post-synaptic mechanisms are responsible for reduced motoneuron excitability (Robinson et al., 1982; Guissard et al., 2001) and we were therefore expecting that, by applying an appropriate methodology, this effect would still be measurable immediately after stretching. However, it is known that pre- and post-synaptic inhibitions in response to conditioning electrical stimuli in both animal experiments (Eccles et al., 1962) and in humans (Kohn et al., 1997) have a very short time course, therefore any inhibitory effect should have already faded away by the time the foot was passively rotated back to testing position (1.5–2 s) leaving “neutral ground” for a different neurological change to be detected. In humans though, not only an inhibition of motoneurons excitability persists throughout the period when the muscle is stretched (Robinson et al., 1982; Etnyre and Abraham, 1986; Voigt and Sinkjær, 1998; Guissard et al., 2001; Hwang, 2002), but also for several seconds (2–15 s) following a single ankle passive dorsiflexion movement (Nielsen et al., 1993, 1995; Hultborn et al., 1996; Wood et al., 1996; Voigt and Sinkjær, 1998). For this reason it has been suggested that this inhibition could be attributed to reduced transmitter release from Ia afferents (post-activation depression) (Nielsen et al., 1995; Hultborn et al., 1996). This however, leads to an inevitable question: if Ia post-activation depression endures up to 15 s following a single dorsiflexion movement, how did we not observe an inhibition in H-reflex immediately after 1 min stretching? In response, the relevance of post-activation depression has been questioned (Voigt and Sinkjær, 1998). Based on the motoneuron excitability recovery behavior in the study by Hultborn et al. (1996) and their own work (Voigt and Sinkjær, 1998), Voigt and Sinkjær (1998) proposed that depression in H-reflex goes through a two-step recovery: a first, fast and more substantial, within 500 ms due to a relief of presynaptic inhibition and a second, slower, related to post-activation depression. Moreover, the longer duration (10–15 s) of the inhibitory effect reported by Nielsen et al. (1995) and Hultborn et al. (1996) compared to that shorter (2–6 s) observed by Voigt and Sinkjær (1998) and Wood et al. (1996) can be attributed to the fact that reflexes were tested after a dorsiflexion movement that brought the ankle in testing position rather than a dorsiflexion movement followed by a plantar flexion movement to return the ankle to testing position (for review see Budini and Tilp, 2016). Taking this evidence together, in our experiment, the inhibitory effect related to the dorsiflexion needed to move the ankle to stretching position and the stretching itself, was likely mostly faded away by the time the foot was rotated back to testing position and the remaining inhibition was possibly counteracted by another opposite effect induced by stretching. It would be meaningful to seek the source of this effect at postsynaptic level because we observed an increase in MEPs. Among the postsynaptic mechanisms both Ib afferents and Renshaw cells feedback inhibition to their homonymous muscles and are at the same time affected by stretching (Matthews, 1933; Fromm et al., 1977). However, Ib afferent do not respond strongly to muscle stretch (Burg et al., 1973; Jami, 1992) making the extent of Ib implication questionable. Also a reduction in recurrent inhibition would not be a completely convincing argument because a diminished Renshaw cells activity would have had a similar result on both H-reflex and MEPs. Nevertheless, as already discussed, we know that some Ia post-activation depression was probably still present up to few seconds following stretching and this would have influenced H-reflex only; in this prospective the hypothesis of reduced recurrent inhibition should not be discarded.

Furthermore, it cannot be excluded that the observed facilitation is to be attributed to the passive plantar flexion movement necessary to reposition the joint from maximal dorsiflexion angle to testing angle. However, the effect of a plantar flexion on H-reflex excitability is controversial with some authors reporting a facilitation in plantar flexion position (Robinson et al., 1982), during (Voigt and Sinkjær, 1998) or after (Wood et al., 1996) a passive plantar flexion movement, and others observing no results (Hultborn et al., 1996; Hwang, 2002).

### MEPs

MEPs in SOL were facilitated up to 2 s following stretching for then quickly return to baseline values. In a previous work we observed a group average increase in MEPs after 2 times 30 s stretching, but this result was not significant (Budini et al., 2017). Guissard et al. (2001) reported that MEPs were inhibited during static stretching but recovered to initial values as soon as the angle at the joint was returned in neutral position. Also in this case the differences could be related to methodological aspects, but contrary to our expectations, we observed a facilitation instead of an inhibition. On the neurological nature of the effect of stretching on MEPs, Guissard et al. (2001) referred to postsynaptic mechanisms acting on excitability at motoneuron level. Others, however, argue against this explanation supporting the hypothesis that the result should be attributed to altered cortical excitability brought about by changes in background firing of spindle afferents (Stuart et al., 2002). Although the methodology applied by Stuart et al. (2002) was different to the one adopted in the present study, the physiological outcome of the conditioning procedure (reduced muscle spindle sensitivity) is comparable. Our results however do not support the suggestion that cortical excitability can be influenced by a variation in discharge rate of muscle spindle afferents because we observed a long lasting inhibition of the T-reflex (Figure 1) in contrast to a very quick recovery of the MEPs (Figure 4). In relation to postsynaptic mechanisms we have no elements to argue for or against the hypothesis of Guissard et al. (2001), but in any case the time course of a possible postsynaptic inhibition induced during stretching would be too short to be detected after stretching. Therefore, another mechanism should be responsible of the observed increase in MEPs amplitude. As discussed for the H-reflex, reduced recurrent inhibition or a modification in tonic Ia afferent activity following the plantar flexion movement required to reposition the joint from maximal dorsiflexion to testing position could be potential explanations.

### Cortical excitability

Post-stretch MEP amplitude transiently increased at the conditioned muscle (SOL), while no amplitude change was found at the control muscle (TA). This result denotes a facilitation along the corticospinal tract of the target muscle. Since there was no change along the corticospinal tract of the control muscle any global increase of corticospinal excitability, such as induced by repeated stretching (30 times), can be excluded. Why the observed post-stretch facilitation in the target muscle has a short-term effect (up to 2 s) remains unclear. To find out whether this facilitation is of spinal and or of intra-cortical origin, further TMS assessments including paired pulse protocols (Kujirai et al., 1993) are required.

### T-reflex

The reduction in T-reflex amplitude following stretching has already been extensively reported in the literature (Guissard et al., 1988; Rosenbaum and Hennig, 1995; Guissard and Duchateau, 2004; Weir et al., 2005; Budini et al., 2017) and therefore, a lack of different responses between the H and the T-reflex was not surprising. In fact, one of the differences between the H- and T-reflex is that only the T-reflex is susceptible to the morphological and mechanical properties of the muscle–tendon unit (Matthews, 1959). During stretching, both intra- and extra-fusal muscle fibers are elongated. When the muscle is then passively returned to its previous length some slack is formed at the muscle spindles polar regions resulting in reduced muscle spindle sensitivity to subsequent changes in length until the slack is regained (Proske et al., 1993; Gregory et al., 1998). Our present work however, has the credit of throwing light on the T-reflex recovery time course showing that although very modest and with a very slow slope, the natural recovery of muscle spindle sensitivity can already be seen within the first 30 s following static stretching. However, it could not be excluded that the recovery was promoted by alpha-gamma co-activation during the elicitation of the reflex itself and/or the TMS stimulation. For this reason it would be interesting to investigate the recovering trend for a longer period after stretching with a longer interval between one tap and the next. It would also be interesting to test whether a strong voluntary contraction or a supra maximal peripheral nerve stimulation would be sufficient for a complete recover.

### Cumulative effect of stretching

In our experimental protocol, volunteers repeated the 30 s stretching for 60 times, but it was not possible to evaluate the cumulative effects of stretching by comparing the first 30 with the last 30 passive stretching because the order of stimulation sequences was randomized. Nevertheless, at the end of the experiment we completed another measurement identical to the one performed at baseline and observed no differences in any of the investigated parameters.

In conclusion, it was confirmed that 1 min static stretching induces a strong and long lasting inhibition of the T-reflex that cannot be attributed to changes in synaptic inhibition. The transient increase in MEPs together with the lack of results on H-reflex would suggest either that a brief facilitation at the post synaptic level may have been concealed by opposite physiological mechanisms at the spinal level or that the facilitation is manifested within supraspinal regions or the corticospinal tract itself.

## Author contributions

FB, EG, MC, DR, and MT: conception or design of the work; FB, EG, MC, DR, PK, and MT: acquisition, analysis, or interpretation of data for the work; FB, EG, MC, DR, PK, and MT: drafting the work or revising it critically for important intellectual content. All authors approved the final version of the manuscript and agree to be accountable for all aspects of the work in ensuring that questions related to the accuracy or integrity of any part of the work are appropriately investigated and resolved. All persons designated as authors qualify for authorship, and all those who qualify for authorship are listed.

### Conflict of interest statement

The authors declare that the research was conducted in the absence of any commercial or financial relationships that could be construed as a potential conflict of interest. The reviewer DGB and handling Editor declared their shared affiliation.
